# Physical activity, obesity and sedentary behaviour and the risks of colon and rectal cancers in the 45 and up study

**DOI:** 10.1186/s12889-018-5225-z

**Published:** 2018-03-06

**Authors:** Carlos Nunez, Visalini Nair-Shalliker, Sam Egger, Freddy Sitas, Adrian Bauman

**Affiliations:** 1 Cancer Research Division, Cancer Council NSW, 153 Dowling St, Woolloomooloo, Sydney, NSW 2011 Australia; 2 Sydney School of Public Health, the University of Sydney, Camperdown, Sydney, NSW 2006 Australia; 3 Faculty of Medicine and Health Science, Macquarie University, Sydney, NSW 2109 Australia

**Keywords:** Physical activity, Obesity, Sedentary behaviour, Colorectal cancer, Prospective study

## Abstract

**Background:**

Obesity and physical activity (PA) are predictors of colon (CC) and rectal (RC) cancers. Prolonged sitting is also emerging as a potential predictor for these cancers. Little knowledge exists about the interactive effects of obesity, PA and prolonged sitting on cancer risk. This analysis assessed independent and interactive effects of PA, body mass index (BMI) and sitting time on CC and RC risks.

**Methods:**

This analysis used data from a prospective study of 226,584 participants aged 45 years and over in New South Wales (NSW), Australia, who joined the 45 and Up study between 2006 and 2009. Baseline data were linked with data relating to mortality, cancer registration, hospital admission and Department of Human Services to December 2010. Multivariable Cox regression was used to estimate adjusted hazard ratios (referred to as relative risks, RRs) and 95% confidence intervals (Cis). Statistical significance was defined as *p* < 0.05.

**Results:**

There were 846 and 369 ascertained cases of CC and RC. BMI was positively associated with CC risk (*p* = 0.003, P-trend = 0.0006) but not with RC. CC risk was increased in participants in the highest BMI quartile (≥29.4-≤50 kg/m^2^) compared to the lowest (15- < 23.6 kg/m^2^), (RR = 1.32, 95% CI:1.08–1.63). PA was associated with CC risk (*p* = 0.02) but not with RC. Specifically, CC risk was lower in individuals partaking in any amount of vigorous activity (time/week) compared to participants with no engagement (RR = 0.78, 95% CI:0.65–0.93). Sitting time was not associated with CC or RC. We found no evidence of interactive effects of PA, BMI and prolonged sitting on cancer risk.

**Conclusion:**

This evidence suggests that a healthy weight and vigorous activity are essential to reduce CC risk since these factors may be independent of each other.

## Background

Colorectal cancer (CRC), defined as cancer of the colon or rectum, is the third most common cancer globally, accounting for almost 1.4 million new cases annually. Considerable variation in its incidence is observed across world regions [1]; the highest reported incidence occurred in Australia and New Zealand (age-standardised rates of 44.8 and 32.2 per 100,000 in men and women respectively) and the lowest in Western Africa (4.5 and 3.8 per 100,000) in 2012 [1]. The burden of CRC increases with the embracement of unhealthy lifestyle choices. Therefore, a substantial number of cases could be preventable by changing these behaviours [2].

High body fatness, defined as having a body mass index (BMI) greater than 25 kg/m^2^, has been classified as a confirmed predictor associated with increased risk of colon and rectal cancers [3, 4]. In Australia, the prevalence of overweight (BMI 25 to 29.9 kg/m^2^) and obesity (BMI ≥30 kg/m^2^) has increased over time from 56% in 1995 to 63% in 2015, being higher in men (70%) than in women (56%) [5]. Conversely, physical activity (PA) has also been established as a predictor associated with a reduced risk of colon cancer (CC), but its association with rectal cancer (RC) remains inconclusive [3]. Physical inactivity, defined as insufficient levels to meet physical activity guidelines http://www.who.int/mediacentre/factsheets/fs385/en/, was estimated to be around 44.5% amongst the adult Australian population during the period 2014–15 [5]. Despite the well-known association of PA with CC, the dose of activity required to diminish risk has not been determined. Australia’s Physical Activity Guidelines for adults currently recommend at least 150 min of moderate activity or 75 min of vigorous activity weekly http://www.health.gov.au/internet/main/publishing.nsf/Content/health-pubhlth-strateg-phys-act-guidelines.

Moreover, interactions between these specific risk factors and cancer risk have been less studied. [6] Some epidemiological evidence suggest that PA has the potential to counteract the detrimental effects of obesity; in those studies, physically active individuals had a reduced risk of cardio-metabolic outcomes regardless of BMI [7]. Two population based case-control studies observed that commuting or leisure time physical activity (LTPA) significantly altered the risk related to a high body fatness on CC although neither study assessed adherence to PA guidelines in negating this effect on cancer risk [8, 9].

Sedentary behavior is not synonymous with physical inactivity [10]. It mostly represents prolonged sitting which is ubiquitous in present-day societies, particularly in high income countries [11]. In recent years, this behaviour has emerged as an additional potential risk factor associated with adverse cardio-metabolic profile, premature mortality and various types of cancer including CRC [12]. The increased cancer risk associated with sedentary behaviour has been found to be independent of PA where the deleterious health consequences of too much sitting persist even after adjusting for the possible confounding effects of PA [10].

The current analysis examined the independent effects of BMI, as a proxy for body fatness, intensity of PA according to guidelines and sitting time on CC and RC risks. We also assessed the interactions between (i) BMI and PA, (ii) sitting time and PA; and (iii) BMI and sitting time, on the risks of developing CC and RC in The Sax Institute’s 45 and Up Study.

## Methods

### Study design, setting and subjects

The Sax Institute’s 45 and Up Study is a prospective, population-based cohort study in the state of New South Wales (NSW), Australia. The study was established to investigate different relationships between a wide range of exposures and health outcomes in the ageing population; details of the study design, sampling method and baseline data collection have been published elsewhere [13]. Eligible participants were randomly sampled from the general population of NSW through the Department of Human Services (formerly Medicare Australia) enrolment database, which provides near complete coverage of the population. A sex-specific baseline questionnaire was mailed to potential study participants who joined the study by completing the questionnaire and signing a consent for routine linkage of their health records to administrative databases. 267,014 men and women aged 45 years and over were recruited between January 2006 and December 2009 [13]. For the purpose of this analysis, we used 45 and Up baseline data and record linkage data from the NSW Cancer Registry (NSWCR), Admitted Patient Data Collection (APDC) which are records of patients’ services provided by hospitals in NSW, Medicare Benefits Schedule (MBS) a list of the Medicare services subsidised by the Australian government, Pharmaceutical Benefits Scheme (PBS) and Registry of Birth Deaths Marriages (RBDM). The 45 and Up Study was approved by the University of New South Wales Human Research Ethics Committee and this analysis was approved by the New South Wales Population and Health Services Research Ethics Committee (HREC/14/CIPHS/54). The use of MBS and PBS data was approved by the Department of Health and Ageing Departmental Ethics Committee.

### Identification of cases

Linkage of the 45 and Up cohort data to the MBS and PBS data was conducted by the Sax Institute, using a unique identifier that was supplied to the Department of Human Services for the acquisition of the respective data. Incident cases of CC and RC and dates of diagnoses were obtained through probabilistic linkage from the NSWCR by the New South Wales Centre for Health Record Linkage (CHeRel) for all cancer registrations until the 31st of December 2010. Notification of new cancer cases is required under the Public Health Act 2010 by pathology laboratories, hospitals, radiotherapy and medical oncology departments. The International Classification of Diseases for Oncology 3rd edition was used to classify incident cases of CC (C18) and RC which included cancers of the rectosigmoid junction (C19–20) [14].

### Data collection

Baseline questionnaire collected self-reported information on age, height, weight, educational attainment, country of birth, medical history, parental history of cancer and personal health behaviours, including: PA, weekly alcohol intake, smoking status, diet and daily time spent sitting.

### Exposure variables

#### Assessment of body mass index

Body mass index, expressed in kg/m^2^, was derived from self-reported weigh (kg) and height (m) at baseline. These questions were phrased as “how tall are you without shoes” and “about how much do you weight”. Consistent with established methods, participants who reported extreme values for height being shorter than 100 cm, taller than 240 cm, weighing ≤35 kg or ≥270 kg, or with a calculated BMI of < 15 kg/m^2^ were excluded from the analysis due to the increased probability of measurement error [15]. All remaining participants were categorised according to baseline BMI quartiles since BMI risk may be more fine grained that the broad World Health Organization (WHO) categories [15]; we examined actual distribution of BMI in the sample as: 15 to < 23.6 (reference), ≥23.6 to < 26.2, ≥26.2 to < 29.4 and ≥29.4 to 50 kg/m^2^. Participants, for whom BMI was not possible to be estimated due to missing values of weight or height, were categorised as “unknown”.

#### Assessment of physical activity

In the baseline questionnaire, PA was measured with items from the Active Australia Survey (AAS) [16], which has been shown to have acceptable reliability and validity [17]. All participants were asked the weekly frequency and time spent on (i) walking, (ii) moderate and (iii) vigorous activity that lasted at least 10 min. Moderate activity included activities such as gentle swimming, social tennis, vigorous gardening or work around the house; while vigorous activity comprised activities that made participants breathe harder or puff and pant such as jogging, cycling, aerobics, competitive tennis, but not household chores or gardening. Time spent on walking and moderate activity were combined as the former is a form of the latter to derive groups according to PA guidelines; zero activity (reference), > 0 to < 150 and ≥150 min/week. Time spent on vigorous activity was categorised as zero activity (reference), > 0 to < 75 and ≥75 min/week. Participants who did not report their activity level were classified as “unknown”.

#### Assessment of sitting time

Sitting time was based on participants’ response to the question “About how many hours in each 24-hour day do you usually spend sitting”. Responses were categorised according to quartiles of the number of hours spent sitting in a 24-h period. The respective quartiles were 0 to < 3 (reference), 3 to < 5, 5 to < 8 or ≥ 8 h/day. Those participants who did not answer this question were classified as “unknown”.

#### Confounders

Potential confounders of CC and RC risks were selected on the basis of published evidence [3] which included: sex (female or male), birth cohorts (1920s, 1930s, 1940s, 1950s or 1960s), educational attainment (no school certificate or other qualifications, school or intermediate certificate, higher school or leaving certificate, trade-apprenticeship, certificate-diploma or university degree-higher), region of birth (Australia or overseas), smoking status (never, former-those who quit smoking 5 year prior baseline or current), weekly alcohol consumption acquired from quartiles of drinkers (0, 1–3, 4–6, 7–13 or ≥ 14 drinks/week), adherence to the Australian guidelines of fruit and vegetable defined as consuming more than 2 serves of fruit and 5 serves of vegetable a day (yes or no), weekly consumption of processed meat based on tertiles (0, 1, or ≥ 2 servings/week), quartiles of weekly intake of red meat (< 2, 2, 3 or ≥ 4 servings/week), weekly consumption of fibre derived from frequency of brown, wholemeal bread and cereal intake (< 7, 7–13, 14–20 or ≥ 21 servings/week), prevalence of diabetes mellitus (yes or no) identified by using diagnostic codes in APDC data, claims for glycosylated haemoglobin (HbA1c) in MBS data and claims for diabetes medication in PBS data (Anatomical Therapeutic Chemical Classification System (ATC) A10) as described in the validation of self-report diabetes and linked data in this cohort [18]; aspirin use (yes or no) outlined as having taken this medication for most of the last 4 weeks, history of colorectal testing (tested less than three 3 years ago, tested more than 3 years ago or never) [19] and parental history of CRC (yes or no).

#### Statistical analysis

Multivariable Cox regression was used to estimate adjusted hazard ratios (referred to as relative risks, RRs) and 95% confidence intervals (Cis). Statistical significance was defined as *p* < 0.05 for BMI, PA or sitting time, using age as the underlying time variable. Participants were censored if they died, were diagnosed with other cancers or were alive at the end of follow up period (31 of December 2010), whichever came first. RRs were estimated for both men and women combined since there was no evidence of effect modification by sex.

We examined potential two-way interactions between BMI, sitting time and PA on CC and RC risks by adding appropriate interaction terms to the models. The proportional hazard assumptions of the Cox regression models were assessed by Wald tests of covariates through log-time interactions. Sensitivity analyses were performed by excluding: (i) participants with BMI < 18.5 kg/m^2^ from the lowest BMI category because weight loss and therefore very low BMI may be due to an undiagnosed cancer; and (ii) removing participants who self-reported poor health status at baseline or those who were diagnosed during the first 6 months to reduce the potential impact of reverse causality. Statistical analyses were performed using SAS software, version 9.4; SAS institute Inc., Cary, NC.

## Results

Of 267,014 baseline questionnaire respondents, 40,430 were excluded from these analyses due to data linkage errors (*n* = 58), having a prevalent cancer other than non-melanocytic skin cancer prior to recruitment (*n* = 24,167) or extreme values for height, weight and/or calculated BMI (*n* = 16,205) [20]. After exclusions, a total of 226,584 participants remained for these analyses. There were 846 incident cases of CC and 369 RC diagnosed between 2006 and December 2010. Mean follow-up was 2.7 years, ranging from 0.0 to 5.5 years. The mean age at recruitment was 62.0 years (age range 45.0 to 106.2 years).

After exclusions, 61.5% of 226,584 reported having a BMI greater than 25 kg/m^2^ with a higher prevalence in men than in women. Of these, 26.1% did not meet guidelines of moderate activity with women being more likely to adhere to guidelines; and 59.0% did not meet guidelines of vigorous activity with men being more likely to achieve 75 min/week. Men were more likely to spend more than 8 h a day on sedentary pursuits than women. Compared to the lowest BMI quartile, the highest quartile was, on average, younger, more socially disadvantaged, Australian born, less likely to be current smoker and more likely to report less healthy eating habits (more likely to consume processed and red meat; and a low level of dietary fibre) and to have been diagnosed as diabetic. Compared to participants who reported sitting < 3 h/day, those who sat 8+ hours/day were younger, least socially disadvantaged, did not adhere to fruit and vegetable guidelines and consumed more processed meat. Participants who adhered to guidelines of moderate PA (≥150 min/week) or vigorous activity (≥75 min/week) compared to those who did not were younger, least socially disadvantaged, less likely to be current smokers and had lower prevalence of diabetes (Tables 1 and 2).

**Table 1:** Characteristics of study participants according to BMI and sitting time

	BMI Kg/m^2^ (%)					Sitting time hours a day (%)				
Characteristic	15- < 23.6	≥23.6- < 26.2	≥26.2- < 29.4	≥29.4-≤50	Unknown	0- < 3	3- < 5	5- < 8	8+	Unknown
total	56,275	54,765	57,130	57,205	1209	31,458	60,710	64,980	52,927	16,509
Male	18,437 (33%)	27,633 (50%)	31,662 (55%)	26,372 (46%)	551 (46%)	13,357 (42.5%)	27,054 (44.6%)	29,959 (46.1%)	27,654 (52.3%)	6631 (40.2%)
Mean Age	65.3	63.6	62.4	61.1	64.9	62.7	63.6	63.6	60.6	67.3
Education										
University degree or higher	5507 (30.5%)	8255 (30.3%)	7991 (25.6%)	5196 (20.0%)	124 (22.9%)	2586 (19.7%)	5302 (19.9%)	7697 (26.0%)	10,437 (38.2%)	1051 (16.5%)
Region of birth										
Australia	12,372 (67.1%)	19,562 (70.8%)	23,510 (74.3%)	20,258 (76.8%)	357 (64.8%)	9779 (73.2%)	19,907 (73.6%)	21,901 (73.1%)	19,725 (71.3%)	4747 (71.6%)
Smoking status										
Current	3187 (17.4)	3480 (12.7%)	3959 (12.6%)	3847 (14.7%)	74 (13.5%)	1937 (14.6%)	3807 (14.2%)	3976 (13.4%)	3746 (13.6%)	1081 (16.4%)
Alcohol consumption (drinks/week)										
14 or more	4467 (24.7%)	7737 (28.4%)	9565 (30.6%)	7734 (29.8%)	131 (24.8%)	3882 (29.6%)	7917 (29.7%)	8534 (28.8%)	7703 (28.1%)	1598 (25.3%)
Adherence to fruit and vegetable guidelines										
No	14,880 (82.1%)	22,133 (81.2%)	25,475 (81.6%)	20,960 (80.7%)	456 (83.4%)	10,586 (80.4%)	21,432 (80.2%)	23,843 (80.6%)	22,956 (84.1%)	5087 (79.4%)
Consumption of processed meat (servings/week)										
2 or more	6412 (34.8%)	10,220 (37.0%)	12,952 (40.9%)	12,144 (46.1%)	217 (39.4%)	5105 (38.2%)	10,722 (39.6%)	12,239 (40.9%)	11,569 (41.8%)	2310 (34.8%)
Intake of red meat (servings/week)										
4 or more	7162 (40.3%)	11,248 (41.9%)	13,770 (44.7%)	12,164 (47.4%)	227 (42.9%)	5549 (42.8%)	11,611 (44.1%)	13,062 (44.7%)	11,713 (43.4%)	2636 (43.2%)
Consumption of fibre (servings/week)										
21 or more	6369 (35.7%)	8818 (32.7%)	8894 (28.8%)	6911 (26.9%)	145 (27.1%)	3902 (30.0%)	8440 (32.0%)	9331 (31.8%)	7700 (28.5%)	1764 (28.7%)
Diabetes Mellitus										
Yes	1546 (8.4%)	2681 (9.7%)	3993 (12.6%)	5956 (22.6%)	134 (24.3%)	1694 (12.7%)	3627 (13.4%)	4141 (13.8%)	3578 (12.9%)	1270 (19.2%)
Aspirin Use										
Yes	4047 (21.9%)	6340 (22.9%)	7861 (24.8%)	7326 (27.8%)	157 (28.5%)	3151 (23.6%)	6828 (25.2%)	7732 (25.8%)	6185 (22.4%)	1835 (27.7%)
History of colorectal testing										
Never	8850 (49.3%)	12,611 (46.7%)	14,467 (46.6%)	12,878 (49.9%)	281 (52.0%)	6432 (49.2%)	12,653 (47.8%)	13,452 (45.8%)	13,296 (49.0%)	3254 (51.7%)
Parental history of CRC										
Yes	1821 (9.9%)	2800 (10.1%)	3280 (10.4%)	2647 (10.0%)	48 (8.7%)	1327 (9.9%)	2698 (10.0%)	3131 (10.5%)	2858 (10.3%)	582 (8.8%)

**Table 2:** Characteristics of study participants according to time spent on moderate and vigorous activity

	Moderate (%)				Vigorous (%)			
Characteristic	0	> 0–149 min	≥ 150 min	Unknown	0	> 0- < 74 min	≥ 75 min	Unknown
Total	11,727	47,304	159,715	7838	98,308	35,293	53,120	39,863
Male	5559 (47.4%)	23,621 (49.9%)	72,004 (45.1%)	3471 (44.3%)	42,526 (43.3%)	18,201 (51.6%)	26,637 (50.1%)	17,291 (43.4%)
Mean Age	64.8	62.2	62.9	65.0	64.0	60.4	59.5	68.2
Education								
University degree or higher	913 (16.9%)	6601 (28.4%)	19,053 (26.8%)	506 (15.3%)	9762 (23.3%)	5820 (32.3%)	8961 (34.0%)	2530 (15.1%)
Region of birth								
Australia	4035 (72.6%)	16,787 (71.1%)	52,792 (73.3%)	2445 (70.4%)	30,882 (72.6%)	13,472 (74.0%)	19,593 (73.6%)	12,112 (70.1%)
Smoking status								
Current	1118 (20.3%)	3498 (14.9%)	9353 (13.1%)	578 (16.8%)	6676 (15.8%)	2273 (12.6%)	3068 (11.6%)	2530 (14,7%)
Alcohol consumption (drinks/week)								
14 or more	1336 (24.6%)	5695 (24.5%)	21,750 (30.5%)	853 (26.2%)	11,703 (27.8%)	5046 (28.0%)	7873 (29.8%)	5012 (30.1%)
Adherence to fruit and vegetable guidelines								
No	4638 (85.0%)	19,632 (84.6%)	56,917 (80.0%)	2717 (81.0%)	34,749 (82.8%)	14,809 (82.5%)	20,929 (79.6%)	13,417 (79.2%)
Consumption of processed meat (servings/week)								
2 or more	2270 (40.8%)	9284 (39.3%)	29,172 (40.5%)	1219 (35.1%)	17,793 (41.8%)	7253 (39.9%)	10,333 (38.8%)	6566 (38.0%)
Intake of red meat (servings/week)								
4 or more	2389 (45.0%)	9583 (41.9%)	31,206 (44.4%)	1393 (43.3%)	18,841 (45.5%)	7709 (43.4%)	10,544 (40.5%)	7477 (45.5%)
Consumption of fibre (servings/week)								
21 or more	1227 (22.9%)	5872 (25.6%)	23,162 (32.9%)	876 (27.3%)	12,375 (29.8%)	5270 (29.6%)	8361 (32.0%)	5131 (31.2%)
Diabetes Mellitus								
Yes	1192 (21.4%)	3572 (15.1%)	9009 (12.5%)	537 (15.5%)	6973 (16.4%)	1878 (10.3%)	2358 (8.9%)	3101 (17.9%)
Aspirin Use								
Yes	1507 (27.1%)	5632 (23.8%)	17,795 (24.7%)	797 (23.0%)	11,351 (26.7%)	3944 (21.7%)	5223 (19.6%)	5213 (30.2%)
History of colorectal testing								
Never	2974 (54.9%)	11,718 (50.9%)	32,586 (46.1%)	1809 (54.8%)	20,141 (48.3%)	8489 (47.5%)	12,515 (47.8%)	7942 (47.5%)
Parental history of CRC								
Yes	524 (9.4%)	2328 (9.8%)	7460 (10.4%)	284 (8.2%)	4171 (9.8%)	1964 (10.8%)	2893 (10.9%)	1568 (9.1%)

Increasing BMI was associated with increased CC risk (*p* = 0.003 and p-trend = 0.0006) (Table 3); participants with a BMI ≥29.4-≤50 kg/m^2^ had a 32% increased CC risk compared to participants with a BMI 15- < 23.6 kg/m^2^ (RR = 1.32, 95% CI: 1.08–1.63). Although diabetes might be an intermediate outcome on the causal pathway between BMI and disease risk [21], further adjustments with diabetes had little influence on the final outcome, thus it was not included in these analyses (data not shown). Sensitivity analysis excluding underweight participants from the lowest BMI group did not appreciably change any effects of relative risk of neither CC nor RC.

**Table 3:** Cox proportional hazards of incident CC according to BMI, sedentary behaviour and types of physical activity

Variable	Events	No person-years	Age-adjusted HR	Multivariable HR^(1)^
BMI Kg/m^2^				
15- < 23.6	195	154,782	1.00	1.00
≥ 23.6- < 26.2	188	150,904	0.99 (0.81–1.21)	0.90 (0.73–1.12)
≥ 26.2- < 29.4	223	156,854	1.13 (0.93–1.37)	1.12 (0.91–1.38)
≥ 29.4-≤50	232	156,109	1.18 (0.98–1.43)	1.32 (1.08–1.63)
Unknown	8	3273	1.94 (0.96–3.94)	2.21 (1.09–4.49)
*P*-value^(a)^				0.003
P-trend^(b)^				0.0006
Sitting hours/day				
0- < 3	105	87,149	1.00	1.00
3- < 5	228	167,892	1.13 (0.90–1.42)	0.98 (0.77–1.25)
5- < 8	267	178,262	1.24 (0.99–1.56)	1.12 (0.88–1.42)
8+	165	143,987	0.95 (0.75–1.22)	1.02 (0.79–1.32)
Unknown	81	44,633	1.51 (1.13–2.02)	0.92 (0.67–1.28)
*P*-value^(a)^				0.55
P-trend^(b)^				0.88
Moderate activity minutes/week				
None	52	30,194	1.00	1.00
> 0–149	175	137,275	0.73 (0.54–1.00)	1.08 (0.76–1.54)
150 or more	583	433,966	0.78 (0.58–1.03)	1.25 (0.90–1.74)
Unknown	36	20,487	1.01 (0.66–1.55)	1.09 (0.65–1.82)
*P*-value^(a)^				0.17
P-trend^(b)^				0.76
Vigorous activity minutes/week				
None	423	270,374	1.00	1.00
Any amount:^(*)^	220	248,354	0.56 (0.48–0.66)	0.78 (0.65–0.93)
> 0–74	97	102,607	0.60 (0.48–0.75)	0.78 (0.61–0.98)
75 or more	123	145,747	0.54 (0.44–0.66)	0.78 (0.63–0.97)
Unknown	203	103,194	1.26 (1.07–1.49)	0.98 (0.81–1.18)
*P*-value^(a)^				0.02
P-trend^(b)^				0.11

^(1)^Multivariable models adjusted for: birth cohort, sex, education, BMI, sitting time, time spent on moderate and vigorous activity, smoking, alcohol, country of birth, guidelines of fruit and vegetables, weekly intake of processed food, red meat and fibre, aspirin, parental history of CRC and history of colorectal testing

^(a)^‘*P*-value’ for each variable corresponds to a test of whether all HRs = 1

^(b)^‘P-trend’ is for test of linear association in the log hazard scale and obtained by substituting the categorical versions of covariates in the Cox model with continuous or ordinal versions where appropriate

‘Unknown’ category was excluded from the estimation of *P*-values and P-trends.

^(*)^The “any amount” category was derived by combining the “> 0–74” and “75 or more” and the HR for “any amount” was estimated by fitting a separate multivariate model

Of the 2 types of activities, only vigorous activity was associated with CC (Table 3). Participants who engaged in any amount of vigorous activity/week had 22% lower risk of developing CC compared to participants who did not perform this type of activity. The respective RR was 0.78 95% CI 0.65–0.93 with no evidence of a dose response relationship.

**Fig. 1:**
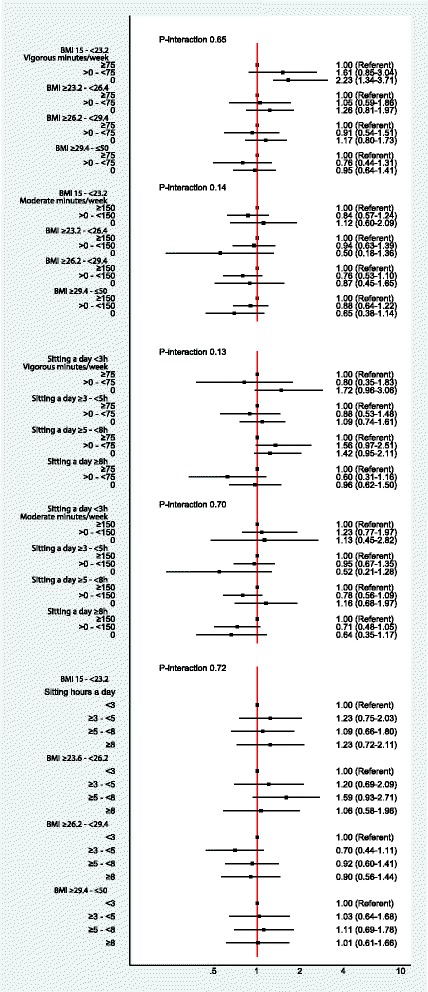
Adjusted hazard ratios and 95% CI for the interaction between BMI-PA, Sitting Time-PA; and BMI-Sitting Time on CC Risk

**Fig. 2:**
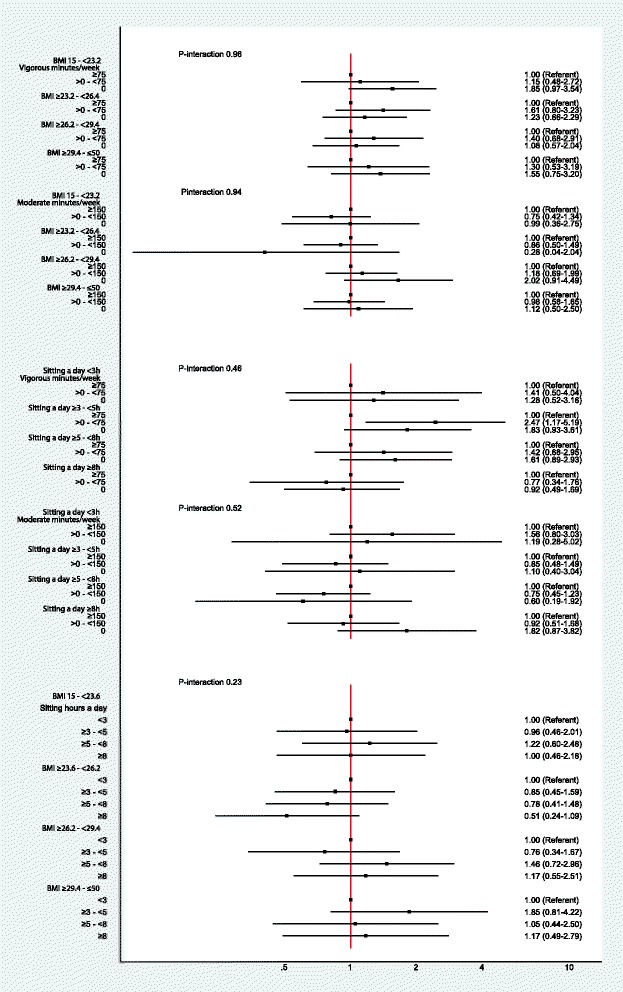
Adjusted hazard ratios and 95% CI for the interaction between BMI-PA, Sitting Time-PA; and BMI-Sitting Time on RC Risk

Risk of CC was not associated with sitting time (*p* = 0.55) or moderate activity (*p* = 0.17) (Table 3). Additionally, RC risk was not associated with BMI (*p* = 0.20), sitting time (*p* = 0.65), moderate activity (*p* = 0.77) or vigorous activity (*p* = 0.11) (Table 4). Sensitivity analysis excluding participants who self-reported poor health status at baseline or those participants who were diagnosed within the first 6 months did not substantially change any effects of relative risks for either CC or RC risk (data not shown). No significant interactions between PA and BMI, sitting time and PA or BMI and sitting time were evident on CC or RC risks in these analyses (*p*-values greater than 0.10) (Figs. 1 and 2).

**Table 4:** Cox proportional hazards of incident rectal cancer according to BMI, sedentary behaviour and types of physical activity

Variable	Events	No person-years	Age-adjusted HR	Multivariable HR^(1)^
BMI Kg/m^2^				
15- < 23.6	93	154,782	1.00	1.00
≥ 23.6- < 26.2	93	150,905	1.03 (0.77–1.37)	0.84 (0.62–1.14)
≥ 26.2- < 29.4	87	156,855	0.92 (0.69–1.24)	0.73 (0.54–1.00)
≥ 29.4-≤50	93	156,110	0.99 (0.74–1.32)	0.76 (0.55–1.04)
Unknown	n.p	3273	1.52 (0.48–4.81)	0.89 (0.22–3.62)
*P*-value^(a)^				0.20
P-trend^(b)^				0.12
Sitting hours/day				
0- < 3	50	87,149	1.00	1.00
3- < 5	100	167,893	1.04 (0.74–1.46)	1.03 (0.72–1.48)
5- < 8	111	178,263	1.09 (0.78–1.52)	1.10 (0.77–1.57)
8+	72	143,988	0.87 (0.61–1.25)	0.90 (0.61–1.32)
Unknown	36	44,633	1.41 (0.92–2.16)	1.07 (0.66–1.74)
*P*-value^(a)^				0.65
P-trend^(b)^				0.40
Moderate activity minutes/week				
None	20	30,195	1.00	1.00
> 0–149	77	102,607	0.85 (0.52–1.39)	0.85 (0.51–1.43)
150 or more	252	433,967	0.88 (0.56–1.38)	0.93 (0.57–1.51)
Unknown	20	20,487	1.47 (0.79–2.73)	1.17 (0.57–2.38)
P-value^(a)^				0.77
P-trend^(b)^				0.21
Vigorous activity minutes/week				
None	163	270,375	1.00	1.00
> 0–74	53	102,607	0.86 (0.63–1.17)	0.98 (0.71–1.36)
75 or more	58	145,748	0.66 (0.49–0.89)	0.70 (0.50–0.98)
Unknown	95	103,194	1.52 (1.18–1.96)	1.34 (1.01–1.79)
*P*-value^(a)^				0.11
P-trend^(b)^				0.78

^(1)^Multivariable models adjusted for: birth cohort, sex, education, BMI, sitting time, time spent on moderate and vigorous activity, smoking, alcohol, country of birth, guidelines of fruit and vegetables, weekly intake of processed food, red meat and fibre, aspirin use, parental history of CRC and history of colorectal testing

^(a)^‘*P*-value’ for each variable corresponds to a test of whether all HRs = 1

^(b)^‘P-trend’ is for test of linear association in the log hazard scale and obtained by substituting the categorical versions of covariates in the Cox model with continuous or ordinal versions where appropriate

‘Unknown’ category was excluded from the estimation of *P*-values and P-trends

n.p Not publishable because of small number, confidentiality or ethical concerns about the data

## Discussion

This is the first analysis to our knowledge that examined the independent and interactive effects of PA based on the Australian recommended guidelines, BMI and sitting time on CC and RC risks. In this Australian cohort, BMI and vigorous activity were independent predictors for CC risk where individuals with a BMI ≥29.4 kg/m^2^ were at higher risk of developing CC than those in the lowest. Also, individuals who engaged in any amount of vigorous activity were at lower risk of developing CC than those who did not partake in this activity. We found no evidence of interactions between any of the study variables assessed and CC or RC risk.

In a recent assessment of observational studies on body fatness and cancer risk, the International Agency for Research on Cancer reported a 30% increased likelihood of developing CRC in those individuals in the highest evaluated BMI compared to the lowest. [4] However, dissociation between colon and rectal cancers was not considered. Some evidence indicate that the aetiology of these two cancers may differ [14]. For instance, a recent meta-analysis of prospective studies observed a 47% increased risk of CC and only 15% for RC when contrasting the highest category of BMI against the lowest [22]. In our analysis, we found that the highest BMI group was similarly associated with an increased risk of CC but not with RC. The lack of association between BMI and RC in our analysis is concordant with a previous prospective Australian study [23].

There is convincing evidence to support a protective role of PA in CC risk. A recent meta-analysis of prospective studies reported a 23% risk reduction for CC when comparing the most active to the least active in the PA spectrum [24]. However, this protective effect of PA on CC risk was based on a combined measure of moderate to vigorous activity (MVPA) without considering the individual type of activity [25–30]. This grouping makes it difficult to determine if intensity and duration are relevant for reducing cancer risk [31]. Very few observational studies have assessed the effects of different types of PA on CC risk [32–35]. A prospective study among Japanese reported a significant risk reduction in CC in men who walked more than 1 h a day, but not in women [35]. Moreover, a prospective cohort documented that vigorous activity decreased CC risk in men but not in women [34]. On the other hand, we found that any amount of vigorous activity reduced CC risk irrespective of sex. This discrepancy in findings might have occurred due to the low number of female cases accrued (fewer than 50) in the studies by Takahashi et al. [35] or Lee et al. [34], while our study had a relatively large number of CC cases for both genders. Also, vigorous physical activity is different from walking as there is a much higher energy expenditure in vigorous activity than walking.

The role of PA on rectal cancer risk is less clear as no association has consistently been observed [32]. A recent meta-analysis of prospective studies reported no change in risk [26]. To our knowledge, there are only three studies that have examined this association by types of PA with RC risk [32, 34, 36]. Of them, only a population based case-control study observed a decreased risk with lifetime vigorous activity [32]. We found no evidence for a relationship between PA and RC risk.

Sedentary behaviour has been proposed as an independent risk factor in colorectal carcinogenesis [10, 37]. A recent meta-analysis of prospective studies reported a 27% increased risk for CC and 6% for RC when comparing the highest amount of sitting to the lowest [37]. However, moderate heterogeneity was observed, reflecting the variability between studies in measuring and categorising this complex behaviour. The different domains of sedentary behaviour include recreation (TV viewing and computer use), workplace sitting and commuting [12]. We did not find any association between total sitting time and the risks of CC or RC nor did we find evidence of two-way interactions with PA or BMI.

The elucidation of the interaction between BMI and PA in terms of cancer risk is of public health interest since these risk factors tend to be related, and have been shown to interact in the context of cardiovascular disease risk [7, 38]. Emerging evidence from case-control studies proposes that PA might offset CC risk related to a high BMI [8, 9]. Only four studies have reported the assessment of the interaction between BMI and PA on CC risk. Of them, two prospective studies reported no significant interactions [14, 27] while two case-control studies detected significant interactions between high levels of lifetime commuting activity or long-term vigorous activity and BMI [8, 9]. We did not observe a significant interaction between PA and BMI on either CC or RC risk. Additionally, our measurement of PA is not comparable to those used in the case-control studies as the questionnaire only assessed current and not lifetime activity. Consistent with our results, we recently reported no evidence of interaction between BMI and current PA on CRC risk in a case-control study [39]. The nature of this interaction does not depend on the scale used as presence on one scale (additive or multiplicative) will also be present on the other scale [40].

Major strengths of this analysis are the prospective nature of the study design, the large cohort sample size of 226,584 participants that provided reasonable statistical power to detect an effect of the exposure variables on cancer risk; and the linkages of the questionnaire data to deaths records, cancer registry and administrative data. A limitation is the relatively short-term follow-up (mean 2.7 years) for cancer incidence which can, depending on the outcome, lead to a low number of cases and imprecise effect estimates. In this study, however, a large number of incident cases for both CC (*n* = 846) and RC (*n* = 369) were accrued during the follow-up period. Another potential limitation is that exposure variables were ascertained by self-report. Nevertheless, BMI and PA have been validated. For instance, a subsample of the 45 and UP cohort showed a strong correlation between self-reported and measured BMI (*r* = 0.95) [20] and the AAS questionnaire possesses a reliability which ranges from 0.56 to 0.64 and validity estimated around 0.52 [41]. Furthermore, vigorous activity tends to be better reported by participants than other categories of recreational PA [41]. Any misclassification of the exposure variables collected before the diagnosis of cancer would most likely have resulted in attenuated estimates of effects [27]. Finally, while we did not find evidence of two-way interactions, confidence intervals for interaction variable categories within were wide, perhaps suggesting limited statistical power to detect such interactions [42].

## Conclusion

This analysis supports the importance of independently adhering to vigorous guidelines of physical activity as well as achieving and maintaining a healthy BMI. Future research using other cohort studies is needed to confirm the absence of interaction between PA and BMI.

## Abbreviations

AAS: Active Australia Survey
APDC: Admitted Patient Data Collection
ATC: Anatomical Therapeutic Chemical Classification System
BMI: body mass index
CC: colon cancer
CHeReL: Centre for Health Record Linkage
CRC: colorectal cancer
LTPA: leisure time physical activity
MBS: Medicare Benefits Schedule
NSW: New South Wales
NSWCR: NSW Cancer Registry
PA: physical activity
PBS: Pharmaceutical Benefits Scheme
RBDM: Registry of Birth Deaths Marriages
RC: rectal cancer
